# Evolutionary relations and population differentiation of *Acipenser gueldenstaedtii* Brandt, *Acipenser persicus* Borodin, and *Acipenser baerii *Brandt

**DOI:** 10.12688/f1000research.10237.2

**Published:** 2016-12-23

**Authors:** Alexey A. Sergeev

**Affiliations:** 1 The Laboratory of Molecular Genetic Identification of Hydrobionts, Russian Federal Research Institute of Fisheries and Oceanography, Moscow, Russian Federation

**Keywords:** Russian Sturgeon, Persian Sturgeon, Siberian sturgeon, AFLP

## Abstract

Russian ( *Acipenser gueldenstaedtii*), Persian ( *A. persicus*) and Siberian ( *A. baerii*) sturgeons are closely related ‘Ponto-Caspian’ species. Investigation of their population structure is an important problem, the solution of which determines measures for conservation of these species. According to previous studies, ‘baerii-like’ mitochondrial genotypes were found in the Caspian Sea among 35% of Russian sturgeon specimens, but were not found in Persian sturgeons. This confirms genetic isolation of the Persian sturgeon from the Russian sturgeon in the Caspian Sea. However, in order to clarify the relationships of these species it is necessary to analyze nuclear DNA markers. The amplified fragment length polymorphism (method) allows estimating interpopulation and interspecific genetic distances using nuclear DNA markers. In the present study, four samples were compared: Persian sturgeons from the South Caspian Sea, Russian sturgeons from the Caspian Sea and the Sea of Azov, and Siberian sturgeons from the Ob’ River, which are close to the latter two species, but are also clearly morphologically and genetically distinct from them. For the amplified fragment length polymorphism (AFLP) method, eight pairs of selective primers were used. The analysis revealed that the Siberian sturgeon has formed a separate branch from the overall Persian-Russian sturgeons cluster, which was an expected result. In addition, the results showed that the Caspian Russian sturgeon is closer to the Persian sturgeon from the Caspian Sea than to the Russian Sturgeon from the Sea of Azov. The present DNA marker data confirm that despite the genetic isolation of the Persian sturgeon from the Russian sturgeon in the Caspian Sea, the Persian sturgeon is a young species.

## Introduction

Three closely related species, the Russian ( *Acipenser gueldenstaedtii*), Persian ( *A. persicus*), and Siberian ( *A. baerii*) sturgeons belong to a polychromosomal group of sturgeon species (2n = 240–260; Vasil’ev, 1985). They form the Ponto–Caspian clade of sturgeons ( Birstein & DeSalle, 1998). *A. persicus* inhabits the Caspian Sea, and *A. gueldenstaedtii* inhabits the Caspian Sea and the Azov Sea ( Berg, 1961). *A. baerii* is geographically isolated from the other two species, and it inhabits Siberian Rivers. Presumably, its Ponto-Caspian ancestors migrated to Siberia ( Birstein & DeSalle, 1998).

These species are closely related, which has caused some difficulties with their molecular genetic identification and clarification of their phylogenetic relations. *A. persicus* was described as a species by Borodin in 1897 ( Borodin, 1897). Later, Berg called it a morphologically distinguishable subspecies of *A. gueldenstaedtii* ( Berg, 1961). Following Berg researchers considered the Persian sturgeon as a subspecies of the Russian sturgeon *Acipenser gueldaenstadti persicus* ( Legeza, 1975), *Acipenser gueldaenstadti persicus natio kurensis* ( Abdurakhmanov, 1962; Legeza & Voinova, 1967). Research of the antigenic components of sturgeon blood serum proteins, carried out in 1974, revealed that the Persian sturgeon is a valid sympatric species ( Lukyanenko *et al.*, 1974a; Lukyanenko *et al.*, 1974b).

The taxonomic rank of *A. persicus* is still disputed. Some researchers point to a distinct morphological differences between Russian and Persian sturgeons ( Artyukhin, 2008; Vasil'eva, 2004). Others find these differences indistinct and point to weakness of mitochondrial DNA marker applying for exact species identification of individuals of Russian, Persian and Adriatic ( *A. naccarii*) sturgeons ( Birstein *et al.*, 2005; Ruban *et al.*, 2008).

The Siberian sturgeon is geographically isolated from the Russian and Persian sturgeons and morphologically is easily distinguishable from them. However, approximately 30% of the Russian sturgeon specimens from the Caspian Sea have mitochondrial DNA that is similar to mitochondrial DNA of *A. baerii* ( Jenneckens *et al.*, 2000). It was shown that a ‘baerii-like’ mitotype of *A. gueldenstaedtii* is similar, but not identical, to mitochondrial DNA of *A. baerii* ( Muge *et al.*, 2008). In total, 2% of Russian sturgeons in the Azov Sea also have a ‘baerii-like’ mitotype ( Timoshkina *et al.*, 2009), whereas this has not been found in Persian sturgeons ( Muge *et al.*, 2008). It is assumed that the ‘baerii-like’ mitochondrial DNA found in some Russian sturgeons from the Caspian Sea is a result of an introgression event during the Pleistocene glaciation ( Muge *et al.*, 2008; Rastorguev *et al.*, 2013).

In order to clarify the phylogenetic relations and population structure of the species within the Ponto-Caspian sturgeon clade, some authors point out the necessity to explore nuclear DNA markers ( Krieger *et al.*, 2008; Miuge *et al.*, 2008). It should be noted that currently researchers have the opportunity to work with single nucleotide polymorphism (SNP) markers, which have been discovered for Ponto-Caspian sturgeons ( Ogden *et al.*, 2013; Rastorguev *et al.*, 2013).

Moreover, to estimate genetic distances within the Ponto-Caspian sturgeon species group, the amplified fragment length polymorphism (AFLP) method is also applicable, as the AFLP technique allows to obtain a high number of dominant nuclear DNA markers ( Congiu *et al.*, 2002). 

By examining the differences between the populations and computing genetic distances we can make suggestions on approximate time of the population separation ( Nei, 1972). The AFLP profiles show patterns of nuclear DNA markers obtained across the whole genome. This data analysis gives an opportunity to estimate genetic similarity of the samples, and statistically verify significance of the differences. However, the method has some limitations. Dominant markers are applicable for polyploid genome studies but less informative than co-dominant markers ( Guillot & Carpentier-Skandalis, 2011). It allows to obtain a large marker set from nuclear DNA but these markers are anonymous ( Vos *et al.*, 1995). We can’t distinguish which of them are selectively neutral and more informative. Therefore, it’s not correct to make the ultimate phylogenetic conclusions based only on this data. The AFLP method could be very useful in comparison with the data obtained from other methods of nuclear DNA marker investigations.

This report presents the results of a molecular genetic study of interpopulation and interspecific genetic distances of the Ponto-Caspian sturgeon clade carried out with the AFLP method.

## Materials and methods

For this research, sturgeon tissue samples (ethanol fixed fin fragments) were obtained from the Russian Federal Reference Collection of Genetic Materials (maintained by the Russian Federal Research Institute of Fisheries and Oceanography, Moscow, Russia). The sample included 24 specimens of *A. gueldenstaedtii* from the Azov Sea (catalog number GUE2906,2908-2930), 24 specimens of *A. gueldenstaedtii* from the Caspian Sea (catalog number GUE2812-2835), 24 specimens of *A. persicus* from the Southern Caspian Sea (catalog number PER0120-143) and 24 specimens of *A. baerii* from the Ob’ River (catalog number BAE0325-348).

DNA was extracted and purified with the Wizard SV Genomic DNA Purification System (Promega). For genetic analysis, the AFLP method was used ( Vos *et al.*, 1995). Briefly, genomic DNA was incubated with the MspI and EcoRI enzyme combination (Fermentas). Next, DNA fragments were ligated with oligonucleotide adapters and used for pre-selective and selective PCR with combinations of fluorescent primers. Selective primer combinations produced sets of markers with different levels of polymorphism. The eight combinations demonstrated the most significant differentiation between samples and were selected for further analysis ( Table 1):

**Table 1.** The list of AFLP primers (Syntol).

PCR	AFLP primers	5’-3’ sequences of oligonucleotides
Pre-selective PCR	ERpr_A Msp_pr_A	gactgcgtaccaattcA gatgagtcctgagcggA
Selective PCR	EcoFAM_AAG EcoFAM_ATT EcoFAM_ACA Msp_pr_AAC Msp_pr_AAG Msp_pr_AAT Msp_pr_ACA Msp_pr_ACC Msp_pr_ACT Msp_pr_ATC	FAM gactgcgtaccaattcAag FAM gactgcgtaccaattcAtt FAM gactgcgtaccaattcAca gatgagtcctgagcggAac gatgagtcctgagcggAag gatgagtcctgagcggAat gatgagtcctgagcggAca gatgagtcctgagcggAcc gatgagtcctgagcggAct gatgagtcctgagcggAtc

1) EcoFAM_AAG - Msp_pr_AAC, 2) EcoFAM_ATT - Msp_pr_AAG, 3) EcoFAM_ACA - Msp_pr_AAT, 4) EcoFAM_AAG - Msp_pr_ACA, 5) EcoFAM_ACA - Msp_pr_ACC, 6) EcoFAM_ATT - Msp_pr_ACC, 7) EcoFAM_AAG - Msp_pr_ACT, 8) Eco-FAM_AAG - Msp_pr_ATC.

Pre-selective PCR was performed for 20 cycles with the following cycle profile: a 30 sec DNA denaturation step at 94°C, a 1 min annealing step at 56°C, and a 1 min extension step at 72°C. Selective PCR was performed for 36 cycles with the following cycle profile: a 30 sec DNA denaturation step at 94°C, a 30 sec annealing step, and a 1 min extension step at 72°C. The annealing temperature in the first cycle was 65°C, was subsequently reduced each cycle by 0.7°C for the next 12 cycles, and was continued at 56°C for the remaining 23 cycles. All steps were carried out with the PTC-225 Peltier Thermal Cycler (MJ Research). 

Capillary electrophoresis was carried out with the ABI Prism Genetic Analyzer 3100 (Applied Biosystems).

Analysis of the obtained AFLP-profiles was performed using Phoretix 1D Advanced v. 5.20 software (Nonlinear Dynamics). The resulting binary matrix was created for further statistical analysis with the program Tools for Population Genetic Analysis v 1.3 (TFPGA). To estimate the allele frequencies of the dominant markers, we used the approach of Lynch & Milligan (1994), which allows work with tetraploid species ( Rodzen & May, 2002). With TFPGA and the unweighted-pair group method with arithmetic means (UPGMA) method, we obtained the matrix of genetic distances ( Nei, 1978) between investigated samples and constructed a dendrogram.

## Results

**Figure 1.**
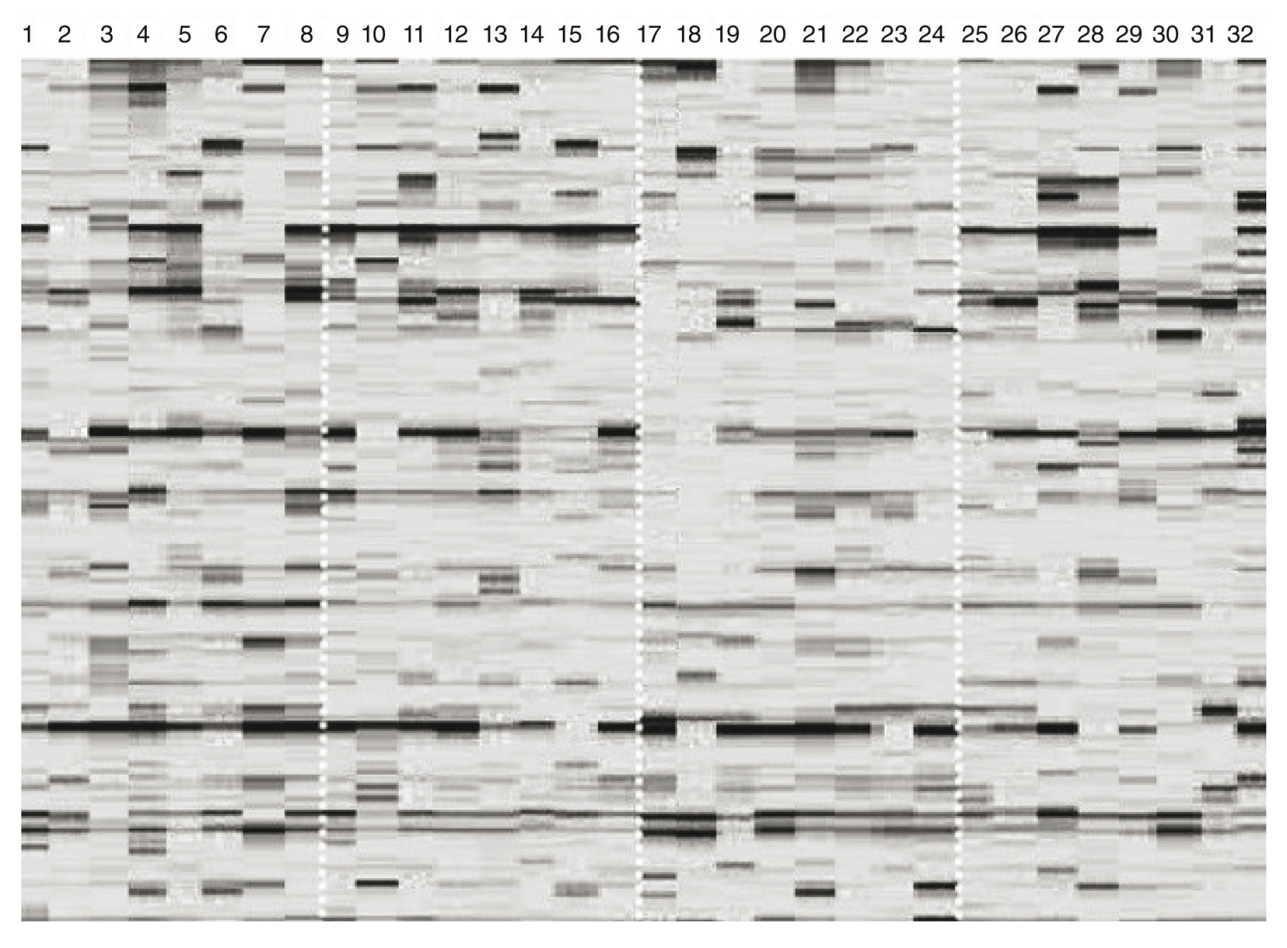
AFLP profile fragments from four sturgeon samples (90-190 bp, EcoFAM_ACA - Msp_pr_ACC primer combination). (1–8) *A. gueldenstaedtii* from the Caspian Sea; (9–16) *A. gueldenstaedtii* from the Azov Sea; (17–24) *A. baerii* from the Ob’ River; and (25–32) *A. persicus* from the Southern Caspian Sea.

Using eight combinations of primers, we obtained AFLP profiles ( Figure 1) with 588 markers (molecular length from 100 to 380 bp). In this study only nuclear DNA markers were investigated. Primary restriction site analysis confirmed that no mitochondrial DNA markers were amplified with used enzymes EcoRI, MspI and the applied primer combinations. In total, 79.59% of the loci were polymorphic. A total of 4 loci were species-specific and monomorphic in the AFLP profiles of *A. baerii*. The differentiation between Russian and Persian sturgeons was observed only in the marker frequencies.

**Figure 2.**
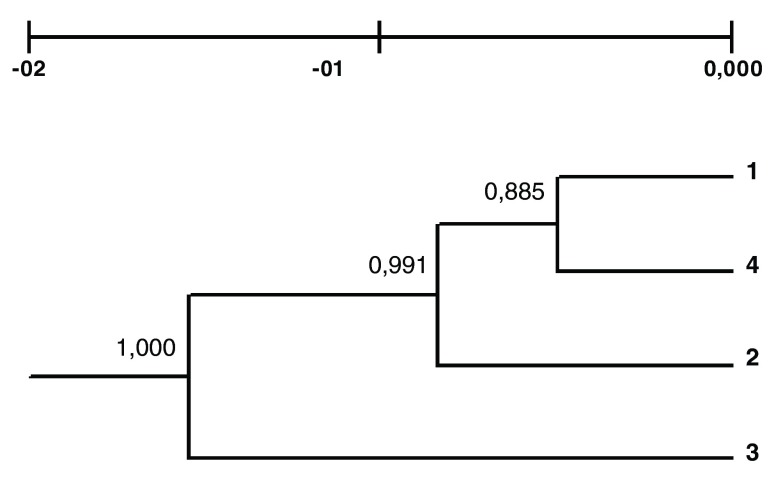
Dendrogram depicting genetic similarity of four sturgeon samples. (1) *A. gueldenstaedtii* from the Caspian Sea; (2) *A. gueldenstaedtii* from the Azov Sea; (3) *A. baerii* from the Ob’ River; and (4) *A. persicus* from the Southern Caspian Sea. Similarities were estimated based on the UPGMA method. The values refer to bootstrap values greater than 0.7.

Using the TFPGA software, genetic distances ( Nei, 1978) were estimated between four sturgeon samples: (1) *A. gueldenstaedtii* from the Caspian Sea; (2) *A. gueldenstaedtii* from the Azov Sea; (3) *A. baerii* from the Ob’ River; and (4) *A. persicus* from the Southern Caspian Sea ( Table 2). We considered the sample size, the amount of obtained markers and used unbiased statistical estimation. The UPGMA dendrogram was constructed with a bootstrap support (1000 permutations) for each node to validate the resulting topology ( Figure 2).

**Table 2.** The matrix of genetic original Nei distances ( Nei, 1978) of four sturgeon samples. (1) *A. gueldenstaedtii* from the Caspian Sea; (2) *A. gueldenstaedtii* from the Azov Sea; (3) *A. baerii* from the Ob’ River; and (4) *A. persicus* from the Southern Caspian Sea.

Sample number	1	2	3	4
1	*****			
2	0.0105	*****		
3	0.0138	0.0213	*****	
4	0.0084	0.0136	0.0224	*****

## Discussion

The AFLP method conducted in the present study revealed that the Siberian sturgeon has formed a branch that is separate from the overall Persian-Russian sturgeon cluster. The Siberian sturgeon is geographically isolated from Persian and Russian sturgeons and is morphologically easily distinguishable from them. According to the results obtained, the Caspian Russian sturgeon is closer to the Persian sturgeon from the Caspian Sea than to the Russian Sturgeon from the Sea of Azov. 

The DNA marker data confirms that, despite the genetic isolation, the Persian sturgeon is a young species. Presumably, the reproductive isolation of Persian sturgeon appeared later than the event of geographic isolation of the Black Sea-Azov and the Caspian basins. Perhaps, there is a gene flow between populations of Persian and Russian sturgeons in the Caspian Sea, which is typical for sturgeons’ natural interspecific hybridization. In this case, it should be mentioned that there is no gene flow from the Russian sturgeon to the Persian sturgeon, as the Persian sturgeon is completely free from the ‘baeri-like’ mitotype, typical for the Russian sturgeon in the Caspian Sea ( Miuge *et al.*, 2008).

The results of this study show the special status of the Russian sturgeon of the Azov Sea, which is geographically and genetically isolated from the Russian sturgeon of the Caspian Sea. This differentiation was shown in previous studies with morphology, mtDNA and STR markers of the Russian sturgeon from the Black Sea-Azov and the Caspian basins ( Timoshkina *et al.*, 2009). The present study has now confirmed these results using the AFLP method.

On the dendrogram, we can observe high bootstrap support values ( Salemi & Vandamme, 2003). However, the obtained genetic distances are unusually small for river spawning species. This can be explained by a slower molecular evolution rate of sturgeons ( Krieger & Fuerst, 2002). Further studies applying SNP and microsatellite analysis approaches are needed in order to confirm results of this study.

## Data availability

The data referenced by this article are under copyright with the following copyright statement: Copyright: © 2016 Sergeev AA

The raw data is available from Zenodo: ( https://zenodo.org/record/167463#.WC8wTtWLTcs) DOI, 10.5281/zenodo.167463 ( Sergeev, 2016).

**Dataset 1** includes AFLP chromatograms (ABI Prism Genetic Analyzer 3100, Applied Biosystems). **Dataset 2** includes AFLP profiles for Phoretix 1D Advanced v. 5.20 software (Nonlinear Dynamics). **Dataset 3** includes TFPGA files (Tools for Population Genetic Analysis v 1.3) with genetic distances and trees.
