# Supplementary material for: Evolutionary relations and population differentiation of Acipenser gueldenstaedtii Brandt, Acipenser persicus Borodin, and Acipenser baerii Brandt
Source: F1000Res. 2016 Dec 23;5:2807. Originally published 2016 Dec 1. [Version 2] doi: 10.12688/f1000research.10237.2 (PMC5147484; doi:10.12688/f1000research.10237.2)
Supplement: Peer review history

# Peer review: Referee response for version 2

**DOI:** 10.5256/f1000research.11321.r18748

**Published:** 2016-12-28

**Authors:** Dmitri D. Pervouchine

**Affiliations:** 1 Bioinformatics and Genomics Programme, Centre for Genomic Regulation (CRG), Barcelona Institute of Science and Technology (BIST), Barcelona, Spain

I have read this submission. I believe that I have an appropriate level of expertise to confirm that it is of an acceptable scientific standard.

# Peer review: Referee response for version 2

**DOI:** 10.5256/f1000research.11321.r18749

**Published:** 2016-12-28

**Authors:** Ekaterina V. Ponomareva

**Affiliations:** 1 Department of Ichthyology, Faculty of Biology, Lomonosov Moscow State University (MSU), Moscow, Russian Federation

I have read this submission. I believe that I have an appropriate level of expertise to confirm that it is of an acceptable scientific standard.

# Peer review: Referee response for version 1

**DOI:** 10.5256/f1000research.11026.r18153

**Published:** 2016-12-08

**Authors:** Dmitri D. Pervouchine

**Affiliations:** 1 Bioinformatics and Genomics Programme, Centre for Genomic Regulation (CRG), Barcelona Institute of Science and Technology (BIST), Barcelona, Spain

The manuscript entitled “Evolutionary relations and population differentiation of *Acipenser gueldenstaedtii* Brandt, *Acipenser persicus* Borodin, and *Acipenser baerii* Brandt” by Alexey Sergeev describes the relationship between mitochondrial genotypes of four closely related sturgeon species by using the amplified fragment length polymorphism (AFPL) method. As a result, an analog of a phylogenetic tree is constructed, one in which the species paradoxically cluster by the habitat ( *A. gueldenstaedtii* from the Caspian Sea is more closely related to *A. persicus* from the Southern Caspian Sea rather than to *A. gueldenstaedtii* from the Azov Sea).

 Generally, the experiment is carried out at a good level and the findings are sufficiently novel. My main criticism is that it is not quite clear to what extent the AFPL method reflects the actual phylogenetic distance between species and that only the mitochondrial genome was interrogated. Generally, the reader has to get an idea of the evolutionary similarity between species by looking at the similarity of patterns in the AFPL profiles. I think the paper can be accepted for indexing if the author explains the caveats and limitations related to this method somewhere in the introduction. Otherwise the readership might be mislead by the dendrogram and the phylogeny reported by the author.

 Currently I have very cosmetic comments which are outlined below.

 Abstract:

 According to previous studies, ‘baerii-like’ mitotypes => mitochondrial genotypes -- the readership might be unfamiliar with this term

 The amplified fragment length polymorphism (method) =>  The amplified fragment length polymorphism method (AFLP) -- the abbreviation must cited in the abstract next to full size sentence, since it appears later without reference

 Persian sturgeons from the South Caspian Sea, Russian sturgeons from the Caspian Sea and the Sea of Azov, and Siberian sturgeons from the Ob’ River, which are close to these - which these? Change 'these' to 'the latter two' - two species, but are also clearly morphologically and genetically distinct from them.

 Main text has to be checked carefully with a native English speaker for word usage.

I have read this submission. I believe that I have an appropriate level of expertise to confirm that it is of an acceptable scientific standard.

# Response

**Published:** 2016-12-14

**Authors:** Alexey Sergeev

**Affiliations:** the Laboratory of Molecular Genetics at the Russian Federal Research Institute of Fisheries and Oceanography (VNIRO), Moscow, Russian Federation

We appreciate a lot such a detailed analysis of the article and very useful comments of Dr. Pervuchine. All suggestions will be applied to the edited version of the article. Some clarifications are needed.

1. In this study the mitochondrial genome was not investigated at all.  It will be mentioned in the article. In fact, mitochondrial DNA of A. gueldenstaedtii has only two restriction sites which EcoRI endonuclease recognizes and about fourty sites matched to MspI. In our research we analyze those fragments of AFLP pattern which meet the following conditions: 1) the AFLP band must have lenghth between 100 – 400 bp, 2) the amplified fragment must be produced with EcoRI selective primer (due to that only EcoRI primer has fluorescent dye), 3) the bases that followed by restriction site should be complementary to the selective bases of primers at final step of amplification. This mtDNA has only two restriction fragments which could be produced but they do not meet these conditions. Previous studies showed weak ability of mitochondrial DNA markers to perform exact species identification of individuals from this sturgeon species group and to clarify their phylogenetic relations. The main goal of this work was to analize nuclear markers as much more informative ones. The AFLP method allows obtaining a large set of anonymous nuclear marker of the genome.
2. AFLP profiles reveal patterns of nuclear DNA markers obtained from the whole genome and reflect its polymorphism. Interrogating these profiles we can estimate similarity of the sample genomes, and statistically verify significance of  AFLP pattern difference which reflect similarity of nuclear genomes. By examining the differences between the populations and computing genetic distances, and taking into account former geological events, we can make suggestions on approximate time of the population and species separation (Nei, 1972).
3. Obviously, this method has some limitations. Dominant markers are applicable for polyploid genome study but less informative than co-dominant markers (G. Guillot and Carpentier-Skandalis, 2010). We obtained large marker set from nuclear DNA but these markers are anonymous. We can’t distinguish which of them are selectively neutral and more informative. We work with them in complex which can somewhat distort the whole picture. Therefore, it’s not correct to make the ultimate phylogenetic conclusions based only on this data. However, these results can be very useful in comparison with the data obtained from other approaches. It will also be mentioned in the article.

 

  Many thanks for very useful comments. They will be very helpful for improving the manuscript.

 1. Guillot G.∗ and Carpentier-Skandalis  А. 2010. On the Informativeness of Dominant and Co-Dominant Genetic Markers for Bayesian Supervised Clustering.  The Open Statistics & Probability Journal 3(1) · December 2011 with 160 ReadsDOI: 10.2174/1876527001103010007

 2. Nei, M. (1972). "Genetic distance between populations". Am. Nat. 106: 283–292. doi:10.1086/282771

# Peer review: Referee response for version 1

**DOI:** 10.5256/f1000research.11026.r18201

**Published:** 2016-12-06

**Authors:** Ekaterina V. Ponomareva

**Affiliations:** 1 Department of Ichthyology, Faculty of Biology, Lomonosov Moscow State University (MSU), Moscow, Russian Federation

In this article, Sergeev addressed the problem having long and complex history of discussion ^1^^-^^3^. Molecular genetic methods (namely, AFLP in Sergeev's study) open a possibility to solve some principal and controversial questions of sturgeon species systematic relationships.

 It is of particular interest also, because the problem has strong practical (and international) dimension, connected with commercial use, conservation and restoration of sturgeons. In spite of significant amount of literature on the subject, there is still a deficit of studies using genomic markers.

 It should be specially mentioned, that Sergeev's study supports differentiation between Russian sturgeons ( *Acipenser gueldenstaedtii*) from the Caspian Sea and the Sea of Azov, previously revealed by using other methods ^4^.

 I think results obtained by Sergeev are important for resolving relationships between *Acipenser *spp.

I have read this submission. I believe that I have an appropriate level of expertise to confirm that it is of an acceptable scientific standard.

## References

[1] BirsteinVRubanGLudwigADoukakisPDeSalleR: The enigmatic Caspian Sea Russian sturgeon: How many cryptic forms does it contain?. *Systematics and Biodiversity*.2005;3(2) : 10.1017/S1477200005001647 203-218 10.1017/S1477200005001647

[2] Vasil'evaED: Morphological data corroborating the assumption of independent origins within octoploid sturgeon species. *J Ichthyology*.2004;44:

[3] KriegerJHettAFuerstPArtyukhinELudwigA: The molecular phylogeny of the order Acipenseriformes revisited. *Journal of Applied Ichthyology*.2008;24(s1) : 10.1111/j.1439-0426.2008.01088.x 36-45 10.1111/j.1439-0426.2008.01088.x

[4] TimoshkinaNNBarmintsevaAEUsatovAVMiugeNS: [Intraspecific genetic polymorphism of Russian sturgeon Acipencer gueldenstaedtii]. *Genetika*.2009;45(9) :1250-9 19824546

# Response

**Published:** 2016-12-14

**Authors:** Alexey Sergeev

**Affiliations:** the Laboratory of Molecular Genetics at the Russian Federal Research Institute of Fisheries and Oceanography (VNIRO), Moscow, Russian Federation

We appreciate the time and effort of Dr. Ponomareva and would like to thank her for detailed analysis of the investigated theme and high appreciation of this work.
